# Parkinson’s disease motor progression in relation to the timing of REM sleep behavior disorder presentation: an exploratory retrospective study

**DOI:** 10.1007/s00702-024-02739-5

**Published:** 2024-01-16

**Authors:** Roberta Bovenzi, Mariangela Pierantozzi, Matteo Conti, Silvia Carignani, Mariana Fernandes, Tommaso Schirinzi, Rocco Cerroni, Nicola Biagio Mercuri, Alessandro Stefani, Claudio Liguori

**Affiliations:** 1 Department of Systems Medicine, University of Rome “Tor Vergata”, Via Montpellier 1, 00133 Rome, Italy; 2 Parkinson’s Disease Unit, University Hospital of Rome “Tor Vergata”, Via Montpellier 1, 00133 Rome, Italy; 3 Sleep Medicine Centre, University Hospital of Rome “Tor Vergata”, Via Montpellier 1, 00133 Rome, Italy

**Keywords:** Parkinson’s disease, REM sleep behavior disorder, Motor progression, H&Y scale, UPDRS, Body first, Brain first

## Abstract

REM sleep behavior disorder (RBD) is a frequent non-motor symptom of Parkinson’s disease (PD), and the timing of its presentation might have a role in the underlying neurodegenerative process. Here, we aimed to define the potential impact of probable RBD (pRBD) on PD motor progression.

We conducted a longitudinal retrospective study on 66 PD patients followed up at the University Hospital of Rome Tor Vergata. Patients were divided into three groups: with post-motor pRBD (pRBD^post^, *n* = 25), without pRBD (pRBD^wo^, *n* = 20), and with pre-motor pRBD (pRBD^pre^, *n* = 21). Hoehn and Yahr (H&Y) scores, Unified PD Rating Scale (UPDRS) motor scores, and levodopa equivalent daily dose were collected at two follow-up visits conducted in a 5-year interval (T0 and T1). pRBD^post^ patients had a greater rate of motor progression in terms of the H&Y scale compared to pRBD^pre^ and pRBD^wo^ patients, without the influence of anti-parkinsonian treatment.

These preliminary findings suggest that the post-motor occurrence of pRBD can be associated with an acceleration in PD motor progression.

## Introduction

Parkinson’s disease (PD) is a neurodegenerative disorder characterized by the widespread accumulation of α-synuclein as a major component of Lewy bodies (LBs), with loss of dopaminergic neurons in substantia nigra pars compacta (SNpc) (Poewe et al. 2017). Along with primary motor features, namely bradykinesia, tremor, and rigidity, which directly result from nigrostriatal dopaminergic degeneration, PD is characterized by a wide range of non-motor symptoms (NMS) related to a heterogeneous impairment of multiple neurotransmitter networks (Schirinzi et al. 2019). NMS, such as REM sleep behavior disorder (RBD), constipation, autonomic dysfunction, neuropsychiatric manifestations, and cognitive disturbances, might appear at different time points throughout the progression of PD, mirroring the trajectory of non-dopaminergic systems involvement (Rocchi et al. 2021; Bellini et al. 2022; Grillo et al. 2022). RBD, which is characterized by the loss of physiological muscle atonia during REM sleep and motor activity associated with dream enactment, represents one of the most frequent NMS of PD, with a medium incidence of 42.3% in all PD patients (Boeve et al. 2007). As well as other NMS, RBD can precede the onset of motor features for more than 20 years (Zhang et al. 2017b) or occur once motor symptoms are already manifest.

It has been hypothesized that the timing of RBD onset may have a role in the specificity of the PD neurodegenerative process (Ferri et al. 2014); still, its contribution to the progression of motor disturbances is far from being defined.

Here, we aimed to investigate the potential role of probable RBD (pRBD) onset in the trajectory of motor progression of the disease. We performed a retrospective analysis on the medical charts of PD patients followed-up over a 5-year follow-up period. By confronting individuals who developed pRBD after the onset of motor symptoms with those with pre-existing, long-standing pRBD, as well as those without pRBD, we aimed to elucidate the potential meaning of RBD timing of manifestation in PD motor progression.

## Materials and methods

### PD population

We conducted a longitudinal retrospective study according to recent guidelines (Vassar and Matthew 2013). The medical charts of 812 PD patients diagnosed with PD according to the 2015 Movement Disorders Society (MDS) diagnostic criteria (Postuma et al. 2015) and followed up at the PD Unit of the University Hospital of Rome Tor Vergata were screened. The main inclusion criteria for this study were: 6-month follow-up visits regularly performed in a time interval of five years (2017–2022); the description of patients’ motor symptoms, anti-parkinsonian drug treatment, and NMS symptoms as reported during at least one follow-up visit (anytime vs. never); the detailed patients’ sleep medical history, also featured by the single-question for identifying the pRBD diagnosis performed at each medical visit (Postuma et al. 2012). Exclusion criteria were: the absence of a detailed sleep-related history, the absence of a bed partner, and an inadequate follow-up period. The medical charts of 118 PD patients meeting these criteria were selected for further analysis. Among these, the medical charts of 25 patients with post-motor onset of pRBD (pRBD^post^) during this 5-year follow-up period were selected for the aim of this study. Then, two groups of patients stratified for sex distribution, age, disease duration, and main clinical parameters at the first medical visit (T0) were randomly selected as controls: 20 patients without pRBD (RBD^wo^) and 21 with pre-motor pRBD (RBD^pre^). For each patient, the main demographic and clinical data were collected at first (T0, 2017) and last (T1, 2022) follow-up visits, including the Hoehn and Yahr (H&Y) stage, the Unified PD Rating Scale (UPDRS) part III score in ON state, and the levodopa equivalent daily dose (LEDD) using the conventional formula (Schade et al. 2020; Cilia et al. 2023). Then, we evaluated and compared the motor progression across the three groups by analyzing changes in the H&Y stage, UPDRS Part III scores, and LEDD from T0 to T1. This study was conducted in accordance with the principles of the Helsinki Declaration. The local ethics committee approved the study.

### Statistical methods

A one-way ANOVA with a post-hoc Tukey HSD test was used to compare the differences in quantitative demographic and clinical variables (age, disease duration, H&Y, UPDRS part III, LEDD) among groups at T0 and T1. Moreover, we calculated the Δ score (T1-T0) of H&Y, UPDRS part III, and LEDD for each subject. Differences of Δ-H&Y, Δ-UPDRS part III, and Δ-LEDD among groups were compared using one-way ANOVA with a post-hoc Tukey HSD test.

A one-way ANOVA was used to compare the differences in quantitative demographic and clinical variables between pRBD^wo^ patients and those with pRBD (pRBD^pre^ + pRBD^post^) as well.

The Chi-square test was used to compare the qualitative (categorical) clinical features between pRBD^wo^, pRBD^pre^, and pRBD^post^ groups and between patients with pRBD^wo^ and patients with pRBD (pRBD^pre^ + pRBD^post^). The statistical significance was set at *p* < 0.05. The statistical analysis was performed in blind with SPSS.

## Results

Twenty pRBD^wo^ patients, 21 pRBD^pre^ patients, and 25 pRBD^post^ patients were included in this observation. Table 1 shows the main demographic and clinical data of the study population.

**Table 1:** The table shows the main clinical and demographic data of the three groups of patients

	Patients without pRBD (*n* = 20)	Patients with post-motor pRBD (*n* = 25)	Patients with pre-motor pRBD (*n* = 21)	*p* value
Sex (M/F)	15/5	15/10	16/5	NS
AAO	57.0 ± 10.5	59.2 ± 9.3	62.0 ± 9.4	NS
Age (T0)	64.2 ± 11.0	66.9 ± 8.4	66.9 ± 8.1	NS
Disease duration (T0)	7.1 ± 4.8	7.6 ± 4.7	4.7 ± 5.0	NS
UPDRS-III scores (T0)	19.2 ± 6.3	20.4 ± 9.0	19.0 ± 9.2	NS
UPDRS-III scores (T1)	25.9 ± 9.4	27.3 ± 11.3	25.3 ± 11.3	NS
Δ-UPDRS-III scores (T1-T0)	6.8 ± 9.2	7.2 ± 6.4	7.0 ± 7.7	NS
H&Y score (T0)	2.0 ± 0.5	2.2 ± 0.5	2.0 ± 0.4	NS
H&Y score (T1)	2.4 ± 0.6	3.0 ± 0.7	2.5 ± 0.5	*p* = 0.002*
Δ-H&Y scores (T1-T0)	0.4 ± 0.5	0.8 ± 0.6	0.4 ± 0.5	*p* = 0.002*
LEDD (T0)	566.7 ± 269.5	616.7 ± 352.4	411.8 ± 256.3	NS
LEDD (T1)	775.8 ± 357.6	898.7 ± 345.3	665.6 ± 386.2	NS
Δ-LEDD scores (T1-T0)	208.9 ± 299.1	284.9 ± 444.6	296.2 ± 481.9	NS
Motor phenotype (RA/TD/M)	9/2/9	7/1/17	6/1/14	NS
Cardiovascular symptoms (Y/N)	3/17	4/21	3/18	NS
Mood disturbances (Y/N)	9/11	14/11	10/11	NS
Visual hallucinations (Y/N)	4/16	6/19	6/15	NS
Cognitive impairment (Y/N)	8/12	4/21	8/13	NS
Gastrointestinal disturbances (Y/N)	5/15	6/19	9/12	NS
Urinary disturbances (Y/N)	6/14	10/15	9/12	NS
Sexual dysfunction (Y/N)	2/18	1/24	1/20	NS
FOG/Falls (Y/N)	9/11	14/11	11/10	NS

Age and disease duration are expressed in years

*n* number; *AAO* age at onset; *UPDRS* Unified Parkinson’s Disease Rating Scale; *RA* rigid-akinetic; *TD* tremor dominant; *M* mixed; *FOG* freezing of gait; *Y* yes; *N* no

*Significant differences between groups using one-way ANOVA. The clinical phenotypes TD, RA, M, were determined by movement disorders specialists based on the predominant motor symptoms observed, namely tremor (TD), bradykinesia and rigidity (RA), or both (M). The main non-motor symptoms were considered those as clinically relevant by movement disorders specialists: cardiovascular symptoms: symptoms of orthostatic hypotension, syncope; mood disturbances: anxiety, depression; gastrointestinal disturbances: constipation, drooling, dysphagia; urinary disturbances: urinary incontinence, obstructive urinary symptoms, nocturia

As expected, the three groups were homogeneous in sex distribution, age, disease duration, and motor and non-motor phenotype; in particular, no differences were found in terms of LEDD, H&Y score, and UPDRS part III scores at T0. At T1, the One-way ANOVA showed a significant difference in the H&Y stage (*p* = 0.002) among the three groups; the post-hoc Tukey HSD analysis allowed us to identify that the pRBD^post^ group differed from the other two groups due to the significantly higher mean H&Y stage (pRBD^post^ vs. pRBD^wo^ *p* = 0.006; PD pRBD^post^ vs. pRBD^pre^ *p* = 0.008). No differences were found between pRBD^wo^ and pRBD^pre^ patients.

The three groups remained homogeneous in terms of both LEDD and UPDRS part III scores from T0 until T1 follow-up visits. No differences were found in terms of Δ-LEDD and Δ-UPDRS part III. A significant difference was found in terms of the Δ-H&Y (*p* = 0.002). The post-hoc Tukey HSD analysis showed that the Δ-H&Y was significantly higher in patients with pRBD^post^ compared to the other two groups (pRBD^post^ vs. pRBD^wo^ *p* = 0.045; PD pRBD^post^ vs. pRBD^pre^ *p* = 0.043).

No differences emerged in main demographic and clinical features, including motor (T0 and T1) and non-motor features (any time) between pRBD^wo^ patients and those with pRBD (pRBD^pre^ + pRBD^post^).

**Fig. 1:**
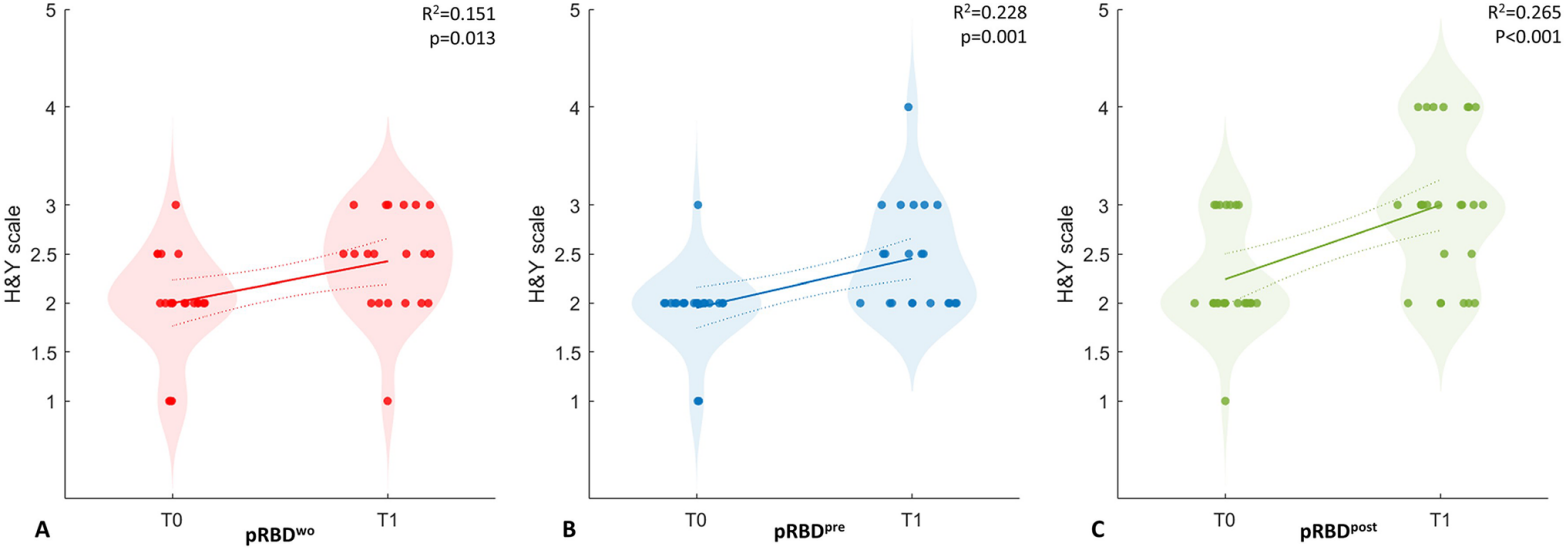
Figure showing motor progression in terms of the H&Y scale score from T0 to T1 (5 year follow-up) in the three groups of patients: patients without pRBD (pRBD^wo^, A), patients with long-standing, pre-motor pRBD (pRBD^pre^, B), and patients with post-motor pRBD (pRBD^post^, C)

Figure 1 shows the progression in the H&Y score from T0 to T1 in the three groups (pRBD^wo^, pRBD^pre^, and pRBD^post^).

## Discussion

The present exploratory study preliminary suggests that PD patients who experienced pRBD following the manifestation of motor symptoms had a faster motor progression compared to those without pRBD and those featured by pre-motor RBD, as demonstrated by the H&Y scale progression over a 5-year follow-up period.

Data on the potential role of RBD onset in PD are rare (Ferri et al. 2014), but they mostly indicate that RBD might influence the clinical presentation of PD; however, no previous study compared the effects of RBD occurrence in the different time points of PD progression.

PD is a heterogeneous syndrome characterized by the misfolding, aggregation, and subsequent cell-to-cell propagation of pathological *α*-synuclein inclusions, which share some similarities with prions (Conti et al. 2022). According to the recent “α-Synuclein Origin and Connectome Model (SOC Model)” of PD, the *α*-synuclein pathology starts in a single location, the body, or the brain, and then propagates symmetrically or asymmetrically, depending on the origin of the accumulation, defining two types of PD: a “brain first” subtype and a “body first” subtype (Grillo et al. 2022; Horsager et al. 2022; Bovenzi et al. 2023; Schirinzi et al. 2023).

RBD can arise in both PD subtypes; as pre-motor RBD in those “body first” subtypes, in which the involvement of pontine and medulla structures precedes the degeneration of mesencephalic dopaminergic nigral cells and the appearance of motor symptoms, and as post-motor RBD in those forms of “brain first” PD, when the descending synucleinopathy originating from the amygdala spreads caudally through the brainstem structures (Horsager et al. 2022).

In either case, RBD reflects the neuronal degeneration or dysfunction in the brainstem regions that regulate the suppression of skeletal muscle tone during REM sleep. Members of this complex circuit include the cholinergic pedunculopontine nucleus (PPN) and lateral dorsal tegmental nucleus (LDTN), the serotonergic raphe nucleus, and glutamatergic and noradrenergic projections from the parabrachial–precoeruleus regions and the locus coeruleus (LC) (Horsager et al. 2022).

Independently from the original site of synucleinopathy, it is now known that the overall burden of specific motor features, such as freezing of gait (FOG), postural instability, and falls, along with several NMS, including constipation, orthostatic hypotension, hyposmia, and cognitive decline, is markedly greater in PD patients with RBD (Assogna et al. 2021; Horsager et al. 2022). Moreover, PD patients with RBD display significantly elevated cerebrospinal fluid prion protein expression levels compared to patients without RBD, identifying a more severe form of neurodegeneration (Zhang et al. 2017a).

In our study, the development of pRBD in PD patients when the motor symptoms were already present was associated with a faster motor progression in a 5-year follow-up period compared to patients without pRBD and patients with pre-motor pRBD.

The finding of greater motor progression in patients with pRBD compared to those without pRBD is in line with the existing literature, indicating RBD as a marker of a more severe neurodegenerative process. However, we found a faster motor progression in patients with post-motor pRBD compared to those with pre-motor pRBD, in apparent contrast with the slower motor progression classically encountered in “brain first” PD. Nevertheless, the faster motor progression in our post-motor pRBD patients in a relatively narrow follow-up period (5 years) may display a particular moment in the track of the neurodegenerative process, in which there is an acceleration of the synucleinopathy in the brainstem areas, with multiple neurotransmitter systems dysfunction leading to the occurrence of sleep disturbances and possible worsening of some motor manifestations. Beyond the dopaminergic circuits, the noradrenergic, cholinergic, and serotonergic systems are deeply involved in PD pathophysiology (Zenuni et al. 2023). In particular, the noradrenergic and cholinergic systems are both involved in the pathophysiology of RBD, as well as postural stability, gait dysfunction, and FOG (Pasquini et al. 2021; Ray Chaudhuri et al. 2023).

The H&Y scale is the most widely used and accepted staging system for the severity of PD. Compared to the UPDRS motor scores, the H&Y scale is more heavily weighted toward some aspects of the disease, such as postural instability and mobility problems, and mixes both the motor impairment and the overall disability (Goetz et al. 2004). Thus, it is possible that the H&Y score, compared to the total UPDRS motor scores, might be more sensitive in detecting the worsening of some motor features mainly driven by noradrenergic and cholinergic network dysfunction, such as gait disturbances and postural instability. However, no significant differences emerged in the occurrence of FOG and falls between the three groups. On the other hand, no differences in terms of LEDD changes over time were observed in the three groups. The reasons underlying this discrepancy cannot be defined here; nevertheless, in advanced stages of PD, the same dosage of levodopa needed to relieve parkinsonian features may also induce motor complications, especially levodopa-induced-dyskinesias, often requiring a reduction in levodopa dosage (Bovenzi et al. 2023). Furthermore, many motor and non-motor symptoms, which arise from non-dopaminergic neurotransmitter dysfunction, including gait and balance dysfunction, are levodopa unresponsive or partially responsive, possibly influencing the amount of LEDD used as the most appropriate. Finally, no differences emerged in the occurrence of main non-motor symptoms, such as cognitive impairment, gastrointestinal disturbances, and cardiovascular symptoms, possibly due to a selection bias in our study population. Indeed, it is possible that a degree of homogeneity in the primary motor features at the first follow-up visit could have influenced the observed non-motor features outcomes.

The main limitations of the present study are the small sample size, although selected from a large group of patients to monitor at the 5-year follow-up, the retrospective design, and the lack of polysomnographic confirmations of RBD.

In conclusion, the present exploratory study suggests that the post-motor occurrence of pRBD may be associated with a fastening of the disease’s motor progression, possibly reflecting a later dysfunction in multiple neurotransmitter networks. Further studies, including larger cohorts or a validation cohort, should deepen the significance of this preliminary observation, also considering the importance of targeting sleep to improve patients’ well-being, as well as motor progression and NMS burden.

## Data Availability

The datasets generated during analysis are available from the corresponding author upon reasonable request.
